# Cloning, Expression, and Functional Characterization of FUT1, a Key Gene for Histo-Blood Group Antigens Synthesis in *Crassostrea gigas*

**DOI:** 10.3390/cimb45050267

**Published:** 2023-05-09

**Authors:** Binbin Gui, Lin Yao, Meng Qu, Weiran Zhang, Mingyu Li, Yanhua Jiang, Lianzhu Wang

**Affiliations:** 1 School of Food Science and Technology, Dalian Polytechnic University, Dalian 116034, China; guibinbingo@163.com; 2 Key Laboratory of Testing and Evaluation for Aquatic Product Safety and Quality, Ministry of Agriculture and Rural Affairs, Qingdao 266071, China; 3 Yellow Sea Fisheries Research Institute, Chinese Academy of Fishery Sciences, Qingdao 266071, China; 4 College of Food Science and Technology, Shanghai Ocean University, Shanghai 201306, China; 5 College of Food Science and Engineering, Ocean University of China, Qingdao 266003, China

**Keywords:** *Crassostrea gigas*, norovirus, HBGAs, gene expression, CgFUT1

## Abstract

Histo-blood group antigens (HBGAs) comprise a family of cell-surface carbohydrates that are considered norovirus-specific binding receptors or ligands. HBGA-like molecules have also been detected in oysters as common norovirus carriers, although the pathway involved in the synthesis of these molecules in oysters has yet to be elucidated. We isolated and identified a key gene involved in the synthesis of HBGA-like molecules, *FUT1*, from *Crassostrea gigas*, named *CgFUT1*. Real-time quantitative polymerase chain reaction analysis showed that *CgFUT1* mRNA was expressed in the mantle, gill, muscle, labellum, and hepatopancreatic tissues of *C. gigas*, with the hepatopancreas exhibiting the highest expression level. A recombinant CgFUT1 protein with a molecular mass of 38.0 kDa was expressed in *Escherichia coli* using a prokaryotic expression vector. A eukaryotic expression plasmid was constructed and transfected into Chinese hamster ovary (CHO) cells. The expression of CgFUT1 and membrane localization of type H-2 HBGA-like molecules in CHO cells were detected using Western blotting and cellular immunofluorescence, respectively. This study indicated that *CgFUT1*, expressed in *C. gigas* tissues, can synthesize type H-2 HBGA-like molecules. This finding provides a new perspective for analyzing the source and synthetic pathway of HBGA-like molecules in oysters.

## 1. Introduction

Noroviruses constitute an important cause of sporadic cases and outbreaks of acute gastroenteritis in humans of all age groups [1,2,3]. Noroviruses are highly contagious, and 10–100 viral particles may be sufficient for infection [4]. Upon entry into the body, noroviruses specifically bind to attachment factors present in the intestine, leading to infection and gastroenteritis [5]. Histo-blood group antigens (HBGAs) have been identified as putative attachment factors for numerous human norovirus strains [6]. Oysters are the largest farmed shellfish in the world, and are farmed around the world. As a filter-feeding bivalve mollusk, oysters typically have the ability to bioaccumulate 100-fold more pathogens than the level present in their environment, which are consequently difficult to remove [7,8]. Thus, they are recognized as common transmission vectors of noroviruses [9]. Multiple studies have reported that the accumulation of noroviruses in oysters is due to their specific binding to carbohydrates present in the digestive ducts of oysters, which have structures similar to human HBGAs [10,11,12]. Therefore, it is reasonable to hypothesize that synthetic pathways and key genes similar to those associated with human HBGA-like molecules may exist in oysters.

In human HBGAs, H antigens produced by fucosyltransferase 1 (FUT1) are believed to serve as ligands during cell adhesion, or as receptors for certain microorganisms such as *Campylobacter jejuni* and *Helicobacter pylori* [13,14,15]. *FUT1* and its homologous gene *FUT2* both express glycosyltransferases that are involved in the synthesis of O(H) blood group antigens; however, these enzymes have different substrate specificities. FUT1 only acts on the type 2 disaccharide precursor Galβ1-4GalNAc, present in blood cells [16]. Most studies regarding the binding of norovirus to tissue blood group antigens report that norovirus binds primarily to secretory blood group antigens, and that individuals with nonsecretory blood groups are resistant to norovirus [17]. In a study that investigated the interactions between saliva from individuals with different blood types and 14 different genotypes of norovirus, 3 genotypes were reported to be able to bind to the saliva of individuals with nonsecretory blood groups, and the rest only bound to the saliva of individuals with secretory blood groups [10,18]. This demonstrates that FUT1 regulates the binding of blood group tissue antigens to noroviruses. Galactose lectin was also found to bind preferentially to H-2 HBGA-like molecules in oysters [19], thereby suggesting that FUT1 plays a role in the innate immunity of oysters.

Although the binding mechanism of HBGAs to norovirus has recently been further elucidated, it remains unclear whether HBGAs in oysters are self-synthesized or derived from foreign microorganisms present in seawater. Herein, *CgFUT1*, a key gene for blood group antigen synthesis in *Crassostrea gigas* (Pacific oyster) tissues, was cloned and confirmed to be immunogenic toward anti-human FUT1 antibody via Western blotting. Using in vitro enzyme function verification and Chinese hamster ovary (CHO) cell transfection assays, CgFUT1 was revealed to exhibit catalytic activity to form H antigen, indicating that this gene may be involved in the H antigen synthesis pathway. Hence, this study provides a theoretical basis for investigating the synthesis pathway of blood group antigens in oysters for the prevention and control of norovirus infections.

## 2. Materials and Methods

### 2.1. Oyster Samples

Fresh samples of *C. gigas* (body length 10–13 cm) were collected from the local shellfish farm (Qingdao, China) and transported on ice. On arrival at the laboratory, within 1 h, five tissue samples were immediately removed from the mantle, gills, muscles, labellum, and hepatopancreas.

### 2.2. Total RNA Extraction and Reverse Transcription

The total RNA from the five tissue of the oysters was prepared using an RNAprep Pure Tissue Kit (catalog No. DP431; TIANGEN, Beijing, China) according to the manufacturer’s instructions. The integrity of the purified RNA was assessed using 1% (*w*/*v*) agarose gel electrophoresis. RNA concentration was determined using a Nano Photometer Pearl microspectrophotometer (Implen, München, Germany). Reverse transcription of the total RNA to synthesize 5′/3′ rapid amplification of cDNA ends (RACE)-ready 1st strand cDNA was performed using a SMATER RACE 5′/3′ kit (catalog No. 634859; Takara, Dalian, China), and cDNA for quantitative real-time polymerase chain reaction (RT-qPCR) analysis was synthesized using an RT-qPCR kit (catalog No. RR047Q; Takara).

### 2.3. Cloning of Full-Length Gene

A pair of PCR primers (FT1A and RT1A; Table 1) was designed using Primer premier 5 (Premier Biosoft, San Francisco, CA, USA) to clone the partial cDNAs of *CgFUT1*. The PCR thermal cycling conditions were as follows: denaturation at 95 °C for 3 min; 35 cycles at 95 °C for 30 s, 48 °C for 30 s, and 72 °C for 1 min; and a final extension at 72 °C for 5 min. To obtain the cDNA 5′ and 3′ ends of *CgFUT1*, two primers (RRT1-A and FRT1-A; Table 1) were designed for 5′ RACE and 3′ RACE, respectively. RACE was performed using a SMATER RACE 5′/3′ kit (Takara), and one 5′ end and 3′ end strip each was obtained. The PCR products were gel-purified and cloned into a pUC19 vector (Takara). Following transformation into competent *Escherichia coli* TOP10, the positive recombinants were identified via antiampicillin selection and PCR screening. Three positive clones were sequenced via Sangon Biotech (Shanghai, China). After sequencing, the 5′ and 3′ ends of the cDNA were ligated using DNAMAN. The full-length cDNA fragments were obtained and named *CgFUT1*.

### 2.4. Sequence and Phylogenetic Analyses

The cDNA and deduced amino acid sequences of *CgFUT1* were analyzed using the online BLAST algorithm (http://blast.ncbi.nlm.nih.gov/Blast.cgi, accessed on 2 March 2022) and ExPASy Translate tool (https://web.expasy.org/translate/, accessed on 2 March 2022). The transmembrane region was predicted using DeepTMHMM (https://dtu.biolib.com/DeepTMHMM, accessed on 2 March 2022). The ExPASy Compute pI/Mw tool (https://web.expasy.org/compute_pi/, accessed on 2 March 2022) was used to compute the physical and chemical parameters of the deduced amino acid sequences. DNAMAN 6 software was used to perform multiple sequence alignment, and a phylogenetic tree was constructed based on the sequence alignment using the neighbor-joining algorithm in MEGA 7.0.

### 2.5. RT-qPCR

*CgFUT1* mRNA expression was analyzed in five tissues of *C. gigas* via RT-qPCR using two primers (Q-FT1-A and Q-RT1-A; Table 1). SYBR Green RT-qPCR assay was performed using the LightCycler^®^ 96 Real-time PCR Detection System (Roche, Basel, Switzerland). *Β-Tubulin*, which is stably expressed in *C. gigas* [20], was selected as the internal reference gene (*F-TUB* and *R-TUB*; Table 1). The RT-qPCR solution was 20 μL, comprising 10 μL SYBR Green Premix Ex Taq, 0.8 μL forward/reverse primer, 2 μL 10× dilution of cDNA template, and 6.4 μL double distilled H_2_O. The cycling conditions were as follows: 95 °C for 3 min, 40 cycles of 95 °C for 5 s, 60 °C for 30 s, and a melting curve analysis from 65 °C to 95 °C. The relative expression of *CgFUT1* in five tissues was calculated using the comparative cycle threshold (2^−ΔΔCt^) method, and the tissue with the lowest expression was used as the basis of measurement. Three biological replicates were available for each tissue [21]. SPSS software 19.0 (IBM SPSS Inc., Chicago, IL, USA) was used for data analysis, and data are represented as means ± standard deviation. Duncan’s multiple range test was used for further analysis. Differences were considered significant if the *p*-value was <0.05.

### 2.6. Expression and Western Blot Analysis of CgFUT1

An *E. coli* codon-optimized DNA sequence containing a 6×His-tag at the C-terminus of *CgFUT1* was commercially synthesized via Genscript (Nanjing, China) and cloned into the pET-28a(+) vector (pET–CgFUT1). Then, the recombinant plasmid pET–CgFUT1 was transformed into *E. coli* BL21 (DE3) cells and incubated overnight at 37 °C. The next day, the overnight culture was inoculated into fresh terrific broth at a 1:50 ratio, with an optical density of 0.5–0.6 at 600 nm; expression was subsequently induced by adding 0.1 mM isopropyl β-d-thiogalactopyranoside (IPTG), following which the culture was incubated for 16 h at 16 °C with gentle shaking. Cells were collected via centrifugation at 6000× *g* for 5 min at 4 °C and washed twice with phosphate-buffered saline (PBS, pH 7.5). The protein sample was separated using 12% sodium dodecyl sulfate–polyacrylamide gel electrophoresis (SDS–PAGE) and transferred to nitrocellulose filter (Merck, Darmstadt, Germany) membranes, which were blocked with 1% bovine serum albumin in TBST (20 mM Tris-HCl, pH 7.4, 150 mM NaCl, and 0.1% Tween-20) for 2 h at room temperature. Membranes were washed with TBST and incubated overnight at 4 °C with anti-6×His-tagged mouse monoclonal antibody at a dilution of 1:3000 (Abcam, Cambridge, MA, USA), or rabbit polyclonal antibody against human FUT1 protein at a dilution of 1:1000 (Abcam). After three washes with TBST, the membranes were incubated in rabbit anti-mouse IgG H&L (HRP) at a dilution of 1:5000 (Abcam), or goat anti-rabbit IgG H&L (HRP) at a dilution of 1:2000 (Abcam), at room temperature. After washing thrice, the films were visualized via Enhanced HRP-DAB Chromogenic Kit (catalog No. PA110; TIANGEN) staining.

### 2.7. Purification of Recombinant Proteins Produced in E. coli

The CgFUT1 recombinant protein was purified using Ni-NTA agarose affinity chromatography (Sangon, Shanghai, China) according to the manufacturer’s instructions. The washing and elution buffer comprised 20 mM Tris-HCl (pH 8.0), 500 mM NaCl, and 8 M urea, along with the following five concentrations of imidazole: 10, 20, 50, and 100 mM for gradient washing, and 250 mM for elution of the target protein. The gravity column was packed with Ni-NTA agarose and equilibrated with 10 mM of imidazole lysis buffer (pH 8.0). The eluent of each concentration gradient was collected and analyzed using SDS–PAGE to confirm the purification of the target proteins.

### 2.8. Validation of the Enzyme Function of CgFUT1

Validation of the enzymatic function was performed following the protocol from Ye et al. [22], with some modifications. A reaction mix comprising 10 μg (2 μL) substrate receptor disaccharide Galβ1-4GlcNAc (Merck), 15 μg (15 μL) UDP-Fuc (QiYue Bio, Xi’an, China), and 25 μL recombinant protein concentrate solution was diluted to 50 μL with buffer (100 mM Tris-HCl, pH 7.5, 20 mM MgCl_2_). A blank control group with no additional recombinant protease, a raw material group containing only recombinant protease solution, and a positive control group with artificially synthesized trisaccharide Fucα1-2Galβ1-4GlcNAc (QiYue Bio) were also prepared. The samples were incubated in a thermostatic bath at 37 °C for 12 h. The reaction was terminated by boiling for 3 min, and the supernatant was centrifuged for thin-layer chromatography (TLC) analysis. The developing solvent used was ethyl acetate:methanol:water:acetic acid (4:2:1:0.2, *v*/*v*/*v*/*v*). The plate was stained using p-anisaldehyde sugar stain.

### 2.9. Expression of CgFUT1 in CHO Cells and Immunofluorescence Assays

A DNA sequence of CgFUT1 containing a 6×His-tag at both the C- and N-terminals was commercially synthesized at Sangon Biotech (Shanghai, China) and cloned into the pcDNA3.1(+) vector (pcDNA3.1–CgFUT1), which was then transfected into CHO cells using Lipofectamine 3000 (Invitrogen, Thermo Fisher, Waltham, MA, USA). The transfected cells were grown in Dulbecco’s Modified Eagle’s Medium (DMEM) containing 10% fetal bovine serum for 48 h. The IF staining followed the protocol described with some modifications [13]. The monolayer cells were washed thrice with PBS at pH 7.3 and fixed at room temperature with precooled 4% paraformaldehyde for 10 min. The cells were divided into two groups, one of which was treated with Triton X-100 0.25% for 10 min to localize the 6×His-tagged CgFUT1 protein. The cells were then washed and treated with 1% bovine serum albumin (BSA) in PBS for 30 min to minimize nonspecific binding. The monolayer was subsequently incubated at room temperature for 90 min with anti-6×His-tagged mouse monoclonal antibody (Abcam) or anti-H-2 mouse monoclonal antibodies (Abcam) at a dilution of 1:30 in PBS with 1% BSA, washed thrice with PBS, and treated with rabbit anti-mouse IgG H&L (Alexa Fluor^®^ 488) (Abcam) or goat anti-mouse IgM H&L (Alexa Fluor^®^ 488) (Abcam) at a dilution of 1:40 in PBS with 1% BSA. The cells were washed thrice with PBS and then incubated with 4′, 6-diamidino-2-phenylindole (DAPI) for 1 min and observed via immunofluorescence microscopy (Leica, Wetzlar, Germany) after PBS rinsing.

## 3. Results

### 3.1. Molecular Characterization of CgFUT1

**Figure 1:**
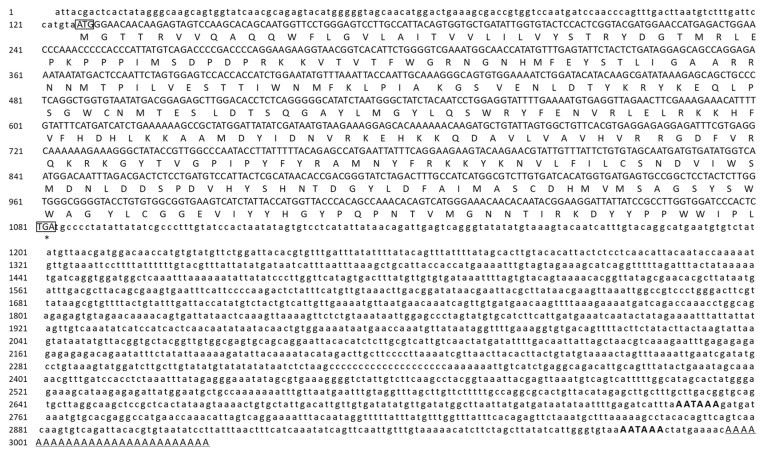
Sequence analysis of *CgFUT1* from *Crassostrea gigas*. Complete nucleotide (above) and predicted amino acid sequence (below) of *CgFUT1* cDNA. The start codon (ATG) and stop codon (TAG) are framed in black. The two polyadenylation signals are shown in bold capital letters, and the poly(A) structure is underlined.

A novel *CgFUT1* gene from *C. gigas* was cloned and characterized using RACE. The complete cDNA sequence of *CgFUT1* was 3024-bp and comprised a 127-bp 5′-untranslated region (UTR), 1940-bp 3′-UTR with a poly(A) tail, and 957-bp open reading frame encoding a predicted polypeptide of 318 amino acids (Figure 1). The deduced amino acid sequence contained a transmembrane region with 20 amino acids (13–32), with a Pfam domain from amino acids 16–319. The theoretical isoelectric point and molecular weight of mature CgFUT1 were 9.51 and 37,189.90 Da, respectively.

### 3.2. Homologous and Phylogenetic Analysis of CgFUT1

**Figure 2:**
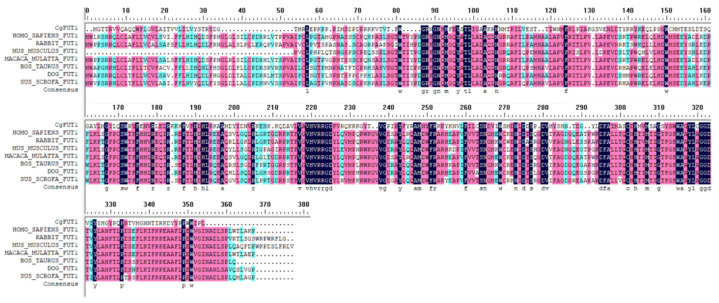
Multiple alignments of amino acid sequences of CgFUT1 with seven different species of FUT1. Identical and similar sites are shown in black and red shades, respectively. Related proteins: *Homo sapiens* NP_000139.1, *Oryctolagus cuniculus* NP_001075872.1, *Mus musculus* NP_001258910.1, *Macaca mulatta* NP_001040619.1, *Bos taurus* NP_803465.2, *Canis lupus familiaris* XP_038512336.1, and *Sus scrofa* NP_999233.1.

**Figure 3:**
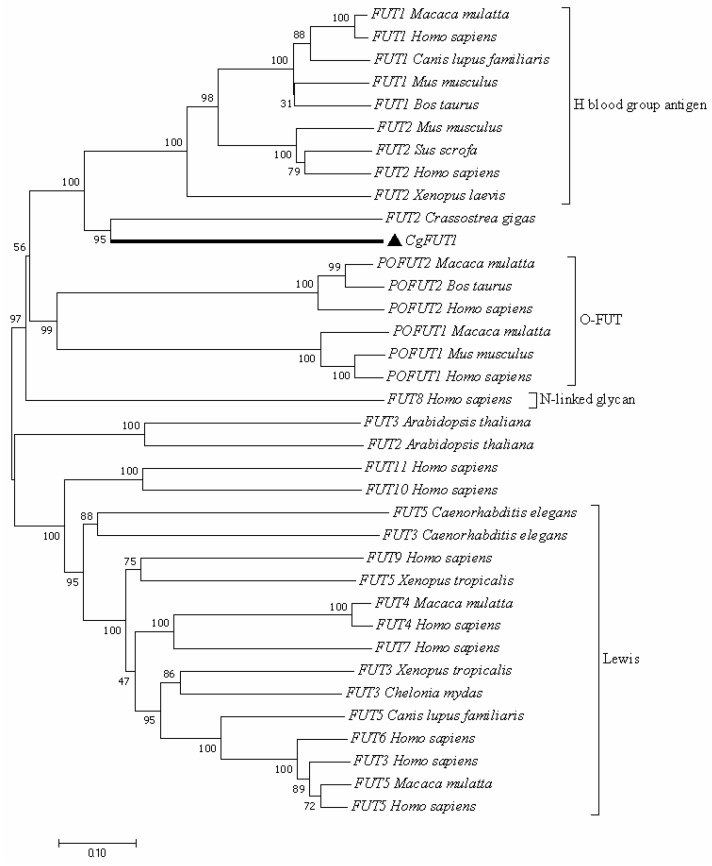
Phylogenetic tree of CgFUT1 sequences. The tree was constructed using the neighbor-joining method in MEGA 7.0, and a bootstrap analysis was performed using 1000 replicates to test the relative support for particular clades. Scale bar signifies an evolutionary distance of 0.1. The following are the Genbank accession numbers of *FUT1*: *Macaca mulatta* NP_001040619.1, *Homo sapiens* NP_000139.1, *Canis lupus familiaris* XP_038512336.1, *Mus musculus* NP_001258910.1, *Bos taurus* NP_803465.2. *FUT2*: *Mus musculus* NP_001258922.1, *Sus scrofa* NP_999234.1, *Homo sapiens* NP_000502.4, *Xenopus laevis* XP_018083932.1, *Crassostrea gigas* XP_011438478.2, *Arabidopsis thaliana* NP_001325360.1. *FUT3*: *Arabidopsis thaliana* NP_177582.3, *Caenorhabditis elegans* NP_495106.1, *Xenopus tropicalis* NP_001004775.1, *Chelonia mydas* XP_007072298.1, *Homo sapiens* NP_000140.1. *FUT4*: *Macaca mulatta* XP_014970974.2, *Homo sapiens* NP_002024.1. *FUT5*: *Xenopus tropicalis* NP_001106419.2, *Caenorhabditis elegans* NP_001022310.1, *Canis lupus familiaris* NP_001005378.1, *Macaca mulatta* XP_014978075.2, *Homo sapiens* NP_002025.2. *FUT6*: *Homo sapiens* NP_000141.1. *FUT7*: *Homo sapiens* NP_004470.1. *FUT8*: *Homo sapiens* NP_001358462.1. *FUT9*: *Homo sapiens* NP_006572.2. *FUT10*: *Homo sapiens* NP_116053.3. *FUT11*: *Homo sapiens* NP_001271123.1. *POFUT1*: *Homo sapiens* NP_056167.1, *Macaca mulatta* XP_028684131.1, *Mus musculus* NP_536711.3. *POFUT2*: *Homo sapiens* NP_056042.1, *Macaca mulatta* XP_028701071.1, and *Bos taurus* NP_991351.1.

Multiple sequence comparisons showed that CgFUT1 has numerous regions of high similarity to other vertebrate FUT1 (Figure 2). A phylogenetic tree was constructed using the FUT genes of different species to characterize the evolutionary relationship of CgFUT1. This showed that CgFUT1 was most similar to FUT1 and FUT2, which express enzymes that synthesize H-2 type antigen in other species (Figure 3).

### 3.3. Tissue Distribution of CgFUT1

**Figure 4:**
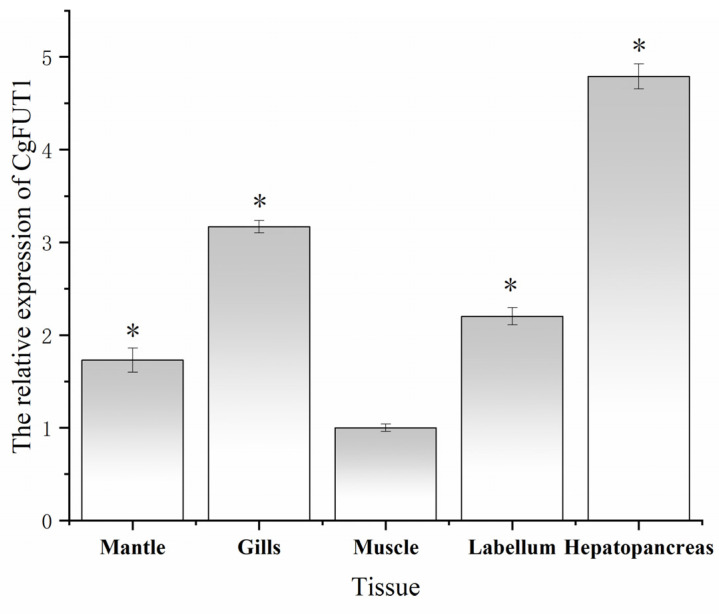
Relative expression of *CgFUT1* mRNA in five *Crassostrea gigas* tissues. * *p* < 0.05 compared with CgFUT1 expression in muscles.

The expression levels of *CgFUT1* mRNA in different *C. gigas* tissues were detected via RT-qPCR. The mRNA transcripts of *CgFUT1* were expressed in the mantle, gill, muscle, labellum, and hepatopancreatic tissues. The lowest level of *CgFUT1* expression was in muscles, and expression in the hepatopancreas was more than four-fold that of muscles. The expression levels in mantle, gill, and labellum tissues were two-to-three-fold that of muscle tissues (*p* < 0.05) (Figure 4).

### 3.4. Expression and Western Blot Analysis of CgFUT1

**Figure 5:**
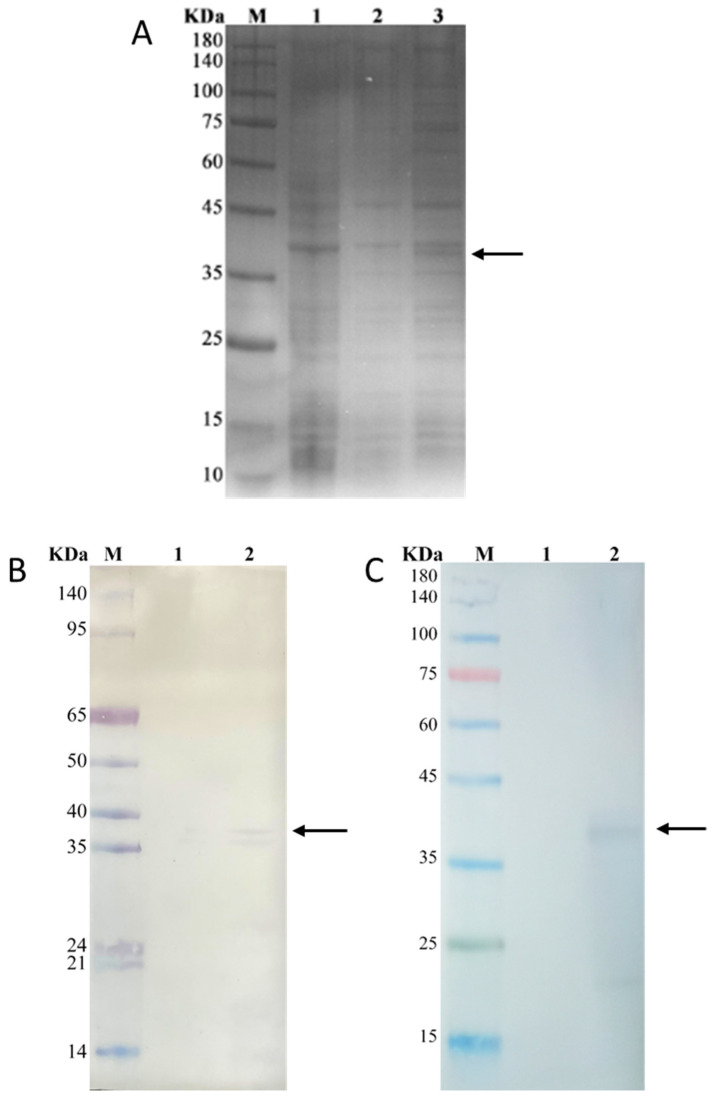
(**A**) CgFUT1 recombinant protein expression was detected via SDS–PAGE. M, protein molecular weight standards; lane 1, cell lysate of *E. coli* BL21 (DE3); lane 2, cell lysate of *E. coli* BL21 (DE3)/pET-28a(+); lane 3, cell lysate of *E. coli* BL21 (DE3)/pET–CgFUT1. (**B**) Western blot analysis of *Escherichia coli* expressing CgFUT1 protein using anti-human FUT1 antibody. M, protein molecular weight standards; lane 1, empty plasmid; lane 2, pcDNA3.1–CgFUT1. (**C**) Western blot analysis of *E. coli* expressing CgFUT1 protein using anti-6×His antibody. M, protein molecular weight standards; lane 1, empty plasmid; lane 2, *E. coli* harboring pET28a–CgFUT1.

The inducible expression of CgFUT1 recombinant protein was performed using pET–CgFUT1. In addition to the expression of the recombinant plasmid, *E. coli* BL21 (DE3) without pET–CgFUT1 and *E. coli* BL21 (DE3) with the pET-28a(+) vector were used as controls. The results showed that a protein band with a molecular size of ~38.0 kDa was observed in the third lane (Figure 5A), which was consistent with the predicted results of the ExPASy tool. Unfortunately, CgFUT1 protein does not seem to be overexpressed in *E. coli*. Subsequently, Western blotting using anti-6×His-tagged mouse monoclonal (Figure 5B) and anti-human FUT1 protein rabbit polyclonal (Figure 5C) antibodies showed a band of ~38.0 kDa in lane 2, whereas lane 1 (controls) showed no band. This result confirmed the successful expression of CgFUT1 after transformation and the similar immunogenicity of recombinant CgFUT1 protein to that of human FUT1 protein.

### 3.5. CgFUT1 Recombinant Protein Purification and Enzyme Function Validation

**Figure 6:**
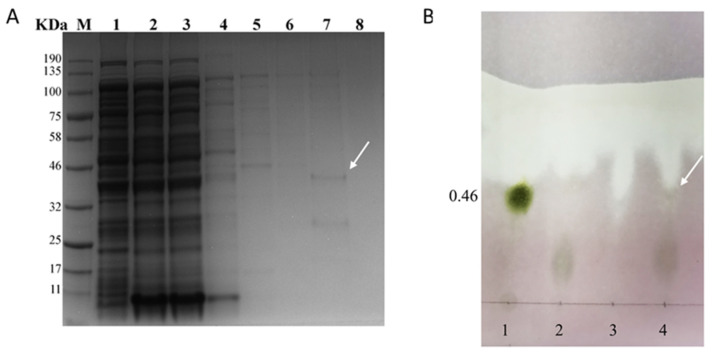
(**A**) SDS–PAGE analysis of purified CgFUT1 protein. M, protein molecular weight standards; lane 1, total protein of induced recombinant bacteria; lane 2, total protein of induced recombinant bacteria in supernatant; lane 3, flow-through liquid; lane 4, 10 mmol imidazole wash fraction; lane 5, 20 mmol imidazole wash fraction; lane 6, 50 mmol imidazole wash fraction; lane 7, 250 mmol imidazole wash fraction; and lane 8, 500 mmol imidazole wash fraction. (**B**) TLC analysis of CgFUT1 recombinant protease activity. Lane 1, Fucα1-2Galβ1-4GlcNAc; lane 2, Galβ1-4GlcNAc+UDP-Fuc; lane 3, CgFUT1 protein solution; and lane 4, reaction mixture of Galβ1-4GlcNAc+UDP-Fuc and CgFUT1 protein.

The CgFUT1 recombinant protein was purified for enzyme function validation using Ni-NTA. SDS–PAGE verification showed that a protein weighing 38.0 kDa was present in the 250 mM imidazole eluent, which was consistent with the size of the pET–CgFUT1 recombinant protein, indicating that the target protein was purified (Figure 6A). The purified product was added to the enzymatic reaction mixture by concentrating and replacing the buffer. TLC of the reaction mixture produced a brown spot at a retention factor (Rf) of ~0.46 on the plate, thereby validating the generation of Fucα1-2Galβ1-4GlcNAc (Figure 6B). This confirmed that the CgFUT1 recombinant protein exhibited the functional activity of α-1, 2-fucosyltransferase.

### 3.6. Expression and Functional Characterization of CgFUT1 in CHO Cells

**Figure 7:**
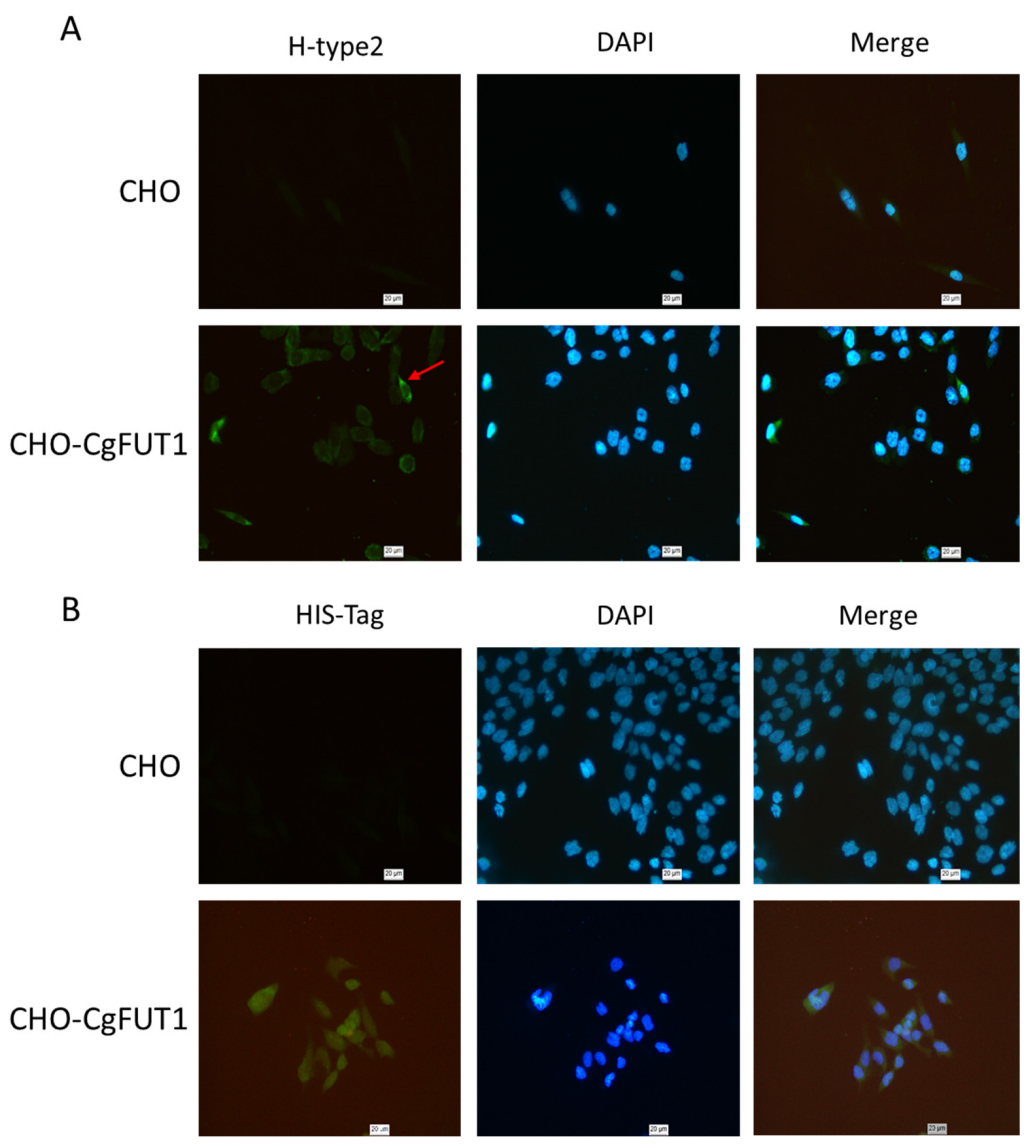
(**A**)Localization of type H-2 HBGAs analyzed via immunofluorescence staining. (**B**) The expression of CgFUT1 protein was analyzed by immunofluorescence staining.

The localization of CgFUT1 (the gene expression product) and H-2 HBGA-like molecules (the catalytic synthesis product) in CHO cells was detected via immunofluorescence (Figure 7), which revealed that the former was mainly present in the cytoplasm, whereas the latter was present in the cell membrane.

## 4. Discussion

Although the interaction and binding mechanism of noroviruses to oyster HBGAs has been previously studied [11,12], there has been little focus on the mode or source of synthesis of oyster tissue blood group antigens. By analyzing the synthesis pathway of human HBGAs, we sought to identify key glycosyltransferase genes in the *C. gigas* genome and verify their functions. We hypothesized that oysters synthesized HBGA-like molecules via a pathway similar to the one occurring in humans.

FUT1, a Golgi glycosyltransferase, is a type II transmembrane enzyme that catalyzes the transfer of monosaccharides to proteins and lipids. FUT1 enzymes share several domain features, such as a single transmembrane domain flanked by a short amino-terminal domain (the so-called cytoplasmic tail) and a large carboxy-terminal catalytic domain oriented toward the lumen of the Golgi apparatus [23]. Multiple sequence alignment showed that CgFUT1 exhibited a high degree of similarity to the vertebrate *FUT1* gene in the C-terminal and several other regions, although the N-terminal sequence (Met-Phe58) exhibited less similarity (Figure 2). This result suggests that the transmembrane structural domain of CgFUT1 is located in the N-terminal region, while the catalytic structural domain begins near Phe58, which is consistent with the structural domain characteristics of Golgi glycosyltransferases. Active glycosyltransferases have been obtained by truncating or replacing their noncatalytic transmembrane structures, N-terminal cytoplasmic tails, and stem regions [24,25,26], which determine the solubility of the glycosyltransferase protein and even the secretion of the glycosyltransferase in eukaryotic cells. This may be the reason why CgFUT1 recombinant proteins cannot be overexpressed in *Escherichia coli.* Therefore, we tried to construct a CgFUT1 plasmid truncated transmembrane domain in the following studies to achieve high expression in *Escherichia coli* and verify this conjecture.

To date, 13 fucosyltransferase genes have been identified in the human genome [27]. As expected, CgFUT1, identified here, is more phylogenetically related to vertebrate FUT1/2, which synthesizes type H-2 blood group antigens, than to FUTs from other species (Figure 3). This indicates that CgFUT1 may synthesize H blood group tissue antigens, consistent with our functional predictions. Notably, phylogenetic analysis of FUT revealed that the development distance of vertebrate FUT1 was longer than that of CgFUT1, which was perhaps due to the large species differences between mollusks and vertebrates. The similarity between FUT1 and FUT2 as a homologous pair of genes would also prevent CgFUT1 from being accurately classified.

In mammals, FUT1 is widely present in various tissues and its catalytic product, H antigen, plays a role in cell adhesion, normal hematopoietic differentiation, and several malignancies, such as colon and pancreas [28]. The distribution of *CgFUT1* mRNA in the five tissues of *C. gigas* was analyzed via RT-qPCR. Our results showed that *CgFUT1* was distributed in all five tissues of *C. gigas*. Furthermore, the relatively high expression of *CgFUT1* in the hepatopancreas is consistent with the fact that blood group tissue antigens are present in the digestive tract of the oyster. A study on the distribution of norovirus in oyster tissues found that the viral load of gill and digestive gland tissue was heavier than that of other tissues [29], which was similar to the tissue distribution of the CgFUT1 gene in this study, indicating that the expression level of the CgFUT1 gene might be correlated with the enrichment of norovirus.

In our study, Western blot analysis of the CgFUT1 recombinant protein revealed two immunoreactive bands near the predicted size of 38.0 kDa for the FUT1 protein (Figure 5B). The experimental conditions were optimized to reduce the impact of the poor specificity of polyclonal antibodies. Given that glycosyltransferases are glycosylated proteins, they can be regarded as potential substrate acceptors for glycosylation, including autoglycosylation. Several studies have also identified FUT1, FUT5, and ST6Gal as capable of autoglycosylation [25,30]. We speculate that autoglycosylation occurs during CgFUT1 expression, resulting in the appearance of two adjacent bands. However, the *E. coli* expression system exhibits a weaker capacity for glycosylation modification than the eukaryotic expression system, and hence, we could not accurately determine whether glycosylation modification occurred during expression in *E. coli*. A study regarding AtFUT1 protein expression reported that the protein was expressed in two forms (tagged and untagged), and it was inferred that protein hydrolysis occurred during the expression [26]. When the CgFUT1 recombinant protein was analyzed via immunoblotting using an anti-6×His-tag antibody, only the larger molecular weight bands were found to bind to the 6×His-tag antibody (Figure 5C), indicating protein hydrolysis may have occurred during the expression of the CgFUT1 protein, resulting in the shedding of the tag.

In vitro enzyme function verification experiments were performed on the purified protein and thin-layer chromatography (TLC) was used to detect the target product in an attempt to determine the glycosyltransferase function of CgFUT1 protein. Unfortunately, fewer Fuc(α1-2)Gal(β1-4)GlcNAc triosaccharide products were detected in TLC analysis (Figure 6B). During CgFUT1 purification, the recombinant CgFUT1 protein could not bind to Ni-NTA packing under nondenaturation conditions, possibly because of the misfolding of the 6×His-tag. However, as protein structure is adversely affected by denaturation, potentially causing the loss of enzyme activity, this may be the reason for the shallow spots of the products in the TLC analysis.

At the same time, considering that secretory and post-translational modifications are the prerequisites for FucTs enzyme activity [31], we suspected that they could not be produced in the prokaryotic host, leading to the poor enzymatic reaction effect of purified proteins in vitro. Therefore, we attempted to express and verify the CgFUT1 gene through eukaryotic cells. As a commonly used eukaryotic expression system, CHO cells do not express α-1,2-amylosyltransferase, a defect that results in a lack of H-2 type antigens on CHO cell membranes [32,33]. In a previous study, human *FUT1* was transfected into CHO cells to study the specific binding of H-2 antigens to campylobacter [13]. Herein, we used cellular immunofluorescence to determine CgFUT1 expression and the localization of the H-2 antigen. As expected, a green fluorescence signal representing the 6×His-tag was mainly concentrated in the cytoplasm (Figure 7B), whereas the green fluorescence signal of the H-2 antigen appeared on the cell membrane (Figure 7A). This finding is consistent with the fact that the FUT1 protein is expressed in the Golgi apparatus and catalyzes the synthesis of blood group tissue antigens on cell membranes.

Structures similar to HBGAs are found in other animals, plants, and even bacteria. Type A and O HBGA-like molecules in the digestive tissues of oysters have been identified as having affinity toward human noroviruses [34,35,36]. These studies also found that several bacteria that express HBGA-like molecules could bind to norovirus particles [35,37,38]. Thus, it remains unclear where the HBGA-like molecules in oysters come from and whether they are synthesized by the oysters or obtained from bacteria present in the oyster gut. *CgFUT1*, cloned in this study, implies the possibility of *C. gigas* having the ability to synthesize HBGA-like molecules.

## 5. Conclusions

In this study, *CgFUT1* of *C. gigas* was successfully cloned and verified as being involved in the synthesis of HBGA-like molecules. This study provides a new perspective for analyzing the source and synthetic pathway of HBGA-like molecules in oysters. Knockout of the CgFUT1 gene in oysters may provide a way to reduce the accumulation of norovirus by oysters in the future.

## Figures and Tables

**Table 1:** Primers used in the study.

Primer	Nucleotide Sequence (5′-3′)	Purpose	Efficiency	Length (bp)
FT1A	AAGCACAGCAATGGTTCC	Cloning partial sequence	–	899
RT1A	CCGTATTGTGTTGTTTCCC			
RRT1-A	GATTACGCCAAGCTTTGGGGGTTTGGGTTCCAGTCTCAT	RACE PCR	–	–
FRT1-A	GATTACGCCAAGCTTTCCTACTCTTGGTGGGCGGGGTA			
Q-FT1-A	CGATGGAACCATGAGACTGGAACC	RT-qPCR	99.0%	141
Q-RT1-A	CTCCTGGCTGCTCCTATCAGAGTAG			
F-TUB	GAGGGTGCTGAACTGGTGG		99.3%	281
R-TUB	CAGAAGGTTTCGTCGGTGT			

## Data Availability

The datasets generated during and/or analyzed during the current study are available from the corresponding author on reasonable request.
